# A Comparative Study of Maternal and Neonatal Outcomes in Obese and Non-obese Iraqi Pregnant Women Undergoing Cesarean Section

**DOI:** 10.7759/cureus.81039

**Published:** 2025-03-23

**Authors:** Salah F Abdulnabi, Mostafa Mohammadi, Ali Jabbari, Somayeh Ghorbani, Omid Nikpayam, Hamed A Flaifel

**Affiliations:** 1 Department of Anesthesiology, Golestan University of Medical Sciences, Gorgan, IRN; 2 Department of Anesthesia Technologies, Alfarqadein University College, Basra, IRQ; 3 Department of Anesthesiology and Intensive Care, Imam Khomeini Hospital Complex, Tehran University of Medical Sciences, Tehran, IRN; 4 Department of Anesthesiology and Critical Care Medicine, Golestan University of Medical Sciences, Gorgan, IRN; 5 Department of Biostatistics and Epidemiology, Golestan University of Medical Sciences, Gorgan, IRN; 6 Department of Nutrition, Golestan University of Medical Sciences, Gorgan, IRN

**Keywords:** cesarean section, maternal outcomes, neonatal outcomes, obesity, pregnancy

## Abstract

Background: Obesity has become a global health concern, and it is on the rise in developed nations among both the general and the obstetric populations. To know if a person has obesity or normal body weight, the body mass index (BMI) is now widely used and regarded as a trustworthy indicator.

Aim: This study was designed to compare maternal and neonatal outcomes between obese and non-obese pregnant women who underwent cesarean section (C/S) in Basra city in the south of Iraq.

Method: This comparative observational cross-sectional study was done in Basra Teaching Hospital, Basra, Iraq, from May 10, 2024, to November 10, 2024, on 206 pregnant women aged between 15 and 50 years old who underwent C/S delivery. The participants were subdivided into three groups according to their BMI: group one: normal weight (n = 64) with a BMI of 18.5-24.9 kg/m², group two: overweight (n = 79) with a BMI of 25-29.9 kg/m², and group three: obese (n = 63) with a BMI of ≥30 kg/m². A study of their preoperative data, including age, blood pressure, BMI, educational level, occupation, gestational hypertension, gestational diabetes mellitus (GDM), previous abortion, previous stillbirth, parity, number of previous C/S, and gestational age, and correlations with their postoperative findings; postpartum hemorrhage (PPH), the time of initial mobility from bed after surgery, the time of starting breastfeeding, the time of starting intestinal sounds, macrosomia, and admission of neonates to the intensive care unit (NICU) was recorded, analyzed, and compared for all three groups. The statistical significance level is considered the p-value < 0.05.

Results: The results demonstrated a clear correlation between maternal and neonatal complications following C/Ss and increased BMI. High BMI was substantially related to maternal factors that differed considerably across the groups, including education level (p-value = 0.001), parity (p-value ≤ 0.001), and the number of previous C/S (p-value = 0.011). Prenatal problems such as GDM (p-value = 0.042) and gestational hypertension (p-value = 0.016) also showed a significant difference, with the obese group experiencing greater rates than the overweight and normal groups. The results showed that there was a significant difference related to maternal postoperative outcomes like the initial time of returning intestinal sounds (p-value = 0.026), the initial time of starting mobility (p-value ≤ 0.001), and the initial time of starting breastfeeding (p-value ≤ 0.001), as well as neonatal postoperative outcomes like macrosomia (p-value = 0.030) and neonatal admission to the NICU (p-value = 0.001); they were also higher in the obese group. Also, the obese group has a longer duration of surgery (p-value ≤ 0.001) and hospital stay (p-value ≤ 0.001) than the other groups. The results showed a non-significant difference regarding PPH among the study groups.

Conclusion: The study concluded that obesity has adverse effects on pregnancy outcomes for mothers and their infants. A low educational level is associated with high BMI, leading to high risks of postoperative complications. Gestational diabetes and gestational hypertension are more frequent in obese women. Obese women should be considered high-risk and recommended to maintain weight to reduce any complications after surgery.

## Introduction

A complicated relationship between sex, societal, environmental, and physiological factors leads to an increase in the risk of obesity [1]. Compared to the male population, women are more likely to be obese, and the possible causes include pregnancy-related weight gain, use of contraceptive pills, estrogen-induced weight gain, lack of social interaction, and physical activity [2]. The worldwide prevalence of overweight and obesity has doubled since 1980 to the extent that nearly a third of the world population is now classified as overweight or obese [3]. Body mass index (BMI) is the most widely used indicator to define obesity, which is calculated by dividing a person's weight in kilograms by the square of his or her height in meters. A person's BMI can be used to determine whether he or she is underweight (BMI < 18.5 kg/m^2^), normal weight (BMI 18.5-24.9 kg/m^2^), overweight (BMI 25.0-29.9 kg/m^2^), or obese (BMI ≥ 30.0 kg/m^2^), and furthermore, obesity is also classified into three levels: class I obesity (BMI 30.0-34.9 kg/m^2^), class II obesity (BMI 35.0-39.9, kg/m^2^), and class III (BMI ≥ 40.0 kg/m^2^) [4]. Pregnancy-related obesity is regarded as a high-risk factor that results in significant fetal-maternal morbidity and mortality [5]. During pregnancy, gestational diabetes mellitus (GDM), preeclampsia, and cesarean section (C/S) are more common among obese women, as well as spontaneous abortion and unexplained stillbirth [6]. Moreover, obesity during pregnancy alters the metabolic programming of the fetus, raising the child's risk of obesity, diabetes, and cardiovascular disease [7]. It is also commonly known that if a mother is overweight or obese relative to a normal weight, there is a greater chance of large-for-gestational-age (LGA) status, macrosomia, admission to the neonatal intensive care unit (NICU), and low Apgar score in the newborn and premature birth [8]. It has also been demonstrated that maternal obesity affects the development of hyperinsulinemia and hypoglycemia during the first few months of a child's life. In the same way, the child has a high chance of developing insulin resistance, hypertension, dyslipidemia, and long-term abnormal body fat distribution. They are also more likely to have behavioral and neuropsychiatric disorders. Statistically, maternally obese women are twice as likely to develop preeclampsia and six times as likely to develop hypertension when compared to pregnant women at an appropriate weight [9]. Also, maternal obesity is related to a higher risk of cesarean deliveries and a higher incidence of anesthetic and postoperative complications [10]. Due to the importance of the pregnancy period and the health status of the neonates, this study aims to compare maternal and neonatal outcomes between obese and non-obese pregnant women who underwent C/S in the south of Iraq.

## Materials and methods

Study design

This comparative descriptive cross-sectional study was done on 206 patients in Basra Teaching Hospital, Basra City, south of Iraq, after getting the approval of the hospital Research Ethics Committee (554 A 7/3/2024) and patient informed consent between May 10, 2024, and November 10, 2024, and in cooperation with Golestan University of Medical Sciences, Gorgan, Iran, after getting the ethical code (IR.GOUMS.REC.1403.006) from the university also.

Study population and selection

The study was performed on pregnant women who were admitted to the obstetric operation room for C/S delivery. Healthy pregnant women (ASA II) with an average age between 15 and 50 years old who were candidates for C/S, had a singleton pregnancy, and were more than 30 weeks along in their pregnancy were included. Women who were pregnant with twins, had Rh mismatches between the mother and baby, or refused to take part in the study were excluded. The participants were subdivided into three groups according to their BMI: group one: normal weight (n = 64) with a BMI of 18.5-24.9 kg/m², group two: overweight (n = 79) with a BMI of 25-29.9 kg/m², and group three: obese (n = 63) with a BMI of ≥ 30 kg/m².

Data collection

A structured checklist by a questionnaire was used to gather the general characteristics to study their preoperative data, including age, blood pressure, BMI, educational level, occupation, gestational hypertension, GDM, previous abortion, previous stillbirth, parity, and the number of previous C/S, and correlations with their postoperative findings; postpartum hemorrhage (PPH), the time of initial mobility from bed after surgery, the time of starting breastfeeding, the time of starting intestinal sounds, macrosomia, and admission of neonates to the NICU were recorded, analyzed, and compared for all three groups.

Statistical analysis

IBM SPSS Statistics for Windows, Version 23 (Released 2015; IBM Corp., Armonk, New York, United States) was used to analyze data. The normality of quantitative variables was assessed using the Kolmogorov-Smirnov test, the normal Q-Q plot, and the histogram of data. Categorical variables were shown by frequency (percentage) and analyzed using the chi-square test. Normally distributed continuous variables were displayed by mean ± SD and analyzed by one-way ANOVA, non-normally distributed quantitative variables by median and quartiles as median (Q1, Q3), and the Kruskal-Wallis test. Simple logistic regression was used to check the effect of variables on neonatal and maternal complications. The statistical significance level is considered the p-value < 0.05.

## Results

In general, 206 pregnant women who underwent C/S weighed 72.21 ± 12.36 kilograms (kg) and had a BMI of 27.85 ± 5.03 kg/m² before pregnancy included in this study. The participants were subdivided into three groups according to their BMI: group one: normal weight (n = 64) with BMI 18.5-24.9 kg/m², group two: overweight (n = 79) with BMI 25-29.9 kg/m², and group three: obese (n = 63) with BMI ≥ 30 kg/m².

The baseline characteristics of participants were classed according to the BMI displayed in Table 1.

**Table 1:** Baseline characteristics of pregnant women based on body mass index category, mean ± SD, median (Q1, Q3), and n (%) ^&^chi-square test; ^*^one-way ANOVA; ^#^Kruskal-Wallis test SD: standard deviation; Q1: first quartile; Q3: third quartile; BMI: body mass index; CS: cesarean section; BPM: beats per minute

Characteristic	BMI			p-value^&^
	Normal weight (n = 64)	Overweight (n = 79)	Obese (n = 63)	
Age (years)	29.80 ± 7.08	31.53 ± 7.27	30.27 ± 6.83	0.314^*^
Occupation n (%)	64 (100.0%)	79 (100.0%)	63 (100.0%)	0.119
Housewife	56 (87.5%)	74 (93.7%)	61 (96.8%)	
Government employee	8 (12.5%)	5 (6.3%)	2 (3.2%)	
Educational level n (%)	64 (100.0%)	79 (100.0%)	63 (100.0%)	0.001
Primary	21 (32.8%)	34 (43.0%)	46 (73.0%)	
Intermediate	28 (43.8%)	29 (36.7%)	10 (15.9%)	
Secondary	6 (9.4%)	8 (10.1%)	3 (4.8%)	
Diploma or higher	9 (14.1%)	8 (10.1%)	4 (6.3%)	
Systolic blood pressure (mmHg)	122.44 ± 10.33	126.27 ± 11.89	131.54 ± 11.73	<0.001^*^
Diastolic blood pressure (mmHg)	73.36 ± 6.63	76.97 ± 9.85	86.19 ± 8.04	<0.001^*^
Heart rate (BPM)	89.25 ± 11.30	90.90 ± 15.15	91.17 ± 14.12	0.689^*^
Parity, median (Q1, Q3)	2 (2, 3)	3 (1, 3)	4 (2, 5)	<0.001^#^
Number of previous C/S, median (Q1, Q3)	1 (0, 2)	1 (0, 2)	2 (1, 3)	0.011^#^
Previous abortion n (%)	64 (100.0%)	79 (100.0%)	63 (100.0%)	0.001
No	54 (84.4%)	70 (88.6%)	40 (63.5%)	
Yes	10 (15.6%)	9 (11.4%)	23 (36.5%)	
Previous stillbirth n (%)	64 (100.0%)	79 (100.0%)	63 (100.0%)	0.004
No	62 (96.9%)	75 (94.9%)	50 (79.4%)	
Yes	2 (3.1%)	4 (5.1%)	13 (20.6%)	
Gestational hypertension n (%)	64 (100.0%)	79 (100.0%)	63 (100.0%)	0.016
No	41 (64.1%)	50 (63.3%)	27 (42.9%)	
Yes	23 (35.9%)	29 (36.7%)	36 (57.1%)	
Gestational diabetes mellitus n (%)	64 (100.0%)	79 (100.0%)	63 (100.0%)	0.042
No	58 (90.6%)	70 (88.6%)	48 (76.2%)	
Yes	6 (9.4%)	9 (11.4%)	15 (23.8%)	

A statistically significant difference was observed among the groups in systolic blood pressure and diastolic blood pressure (p-value < 0.001 for both variables). The mean and standard deviation of systolic and diastolic blood pressure in the obese group were 131.54 ± 11.73 and 86.19 ± 8.04, respectively, higher than in overweight and normal weight groups. The results also showed that there was a statistically significant difference among the groups in the parity (p-value < 0.001) and the number of previous C/S (p-value = 0.011). The median number of parity 4 (2, 5) and the median number of previous C/S 2 (1, 3) were higher in the obese group than in the other two groups.

In addition, there was a statistically significant difference among groups in educational level (p-value = 0.001). The number of pregnancies that had primary school level (46, 73.0%) in the obese group was the highest among the groups. The number of pregnant women who had secondary and intermediate school levels was 8 (10.1%) and 29 (36.7%), respectively, higher in the overweight group in comparison to others. However, the number of graduated levels (diploma or higher) was (9, 14.1%) the highest in the normal weight group.

Additionally, there was a statistically significant difference concerning previous abortion (p-value = 0.001) and previous stillbirth (p-value = 0.004). In the obese group, the number of previous abortions (23, 36.5%) and the number of previous stillbirths (13, 20.6%) was higher compared to the normal weight and the overweight groups.

Furthermore, there was a statistically significant difference in gestational hypertension (p-value = 0.016) and GDM (p-value = 0.042). The number of pregnant women with gestational hypertension (36, 57.1%) and the number of pregnant women with GDM (15, 23.8%) were higher in the obese group than in the other groups.

However, there was no statistically significant difference among groups in other variables, including age, occupation, and heart rate.

Table 2 showed that there was a statistically significant difference among groups regarding maternal postoperative outcomes, including the initial time of starting mobility from bed (p-value < 0.001), the initial time of returning intestinal sounds (p-value = 0.026), duration of surgery (p-value < 0.001), the initial time of starting breastfeeding after surgery (p-value < 0.001), and length of stay in the hospital after surgery (p-value < 0.001). Also, the results showed that there was a statistically significant difference among groups regarding neonatal postoperative outcomes, including macrosomia (p-value = 0.030) and the admission of the neonates into the NICU (p-value = 0.001). All of them were higher in the obese group than the other groups; where the median of the initial time of starting mobility from bed in the obese group was 7 (7, 9) hours, the median of the initial time of returning intestinal sounds in the obese group was 10 (8, 14), the median of the duration of surgery in the obese group was 45 (40, 60) minutes, the median of the initial time of starting breastfeeding in the obese group was 8 (4, 12) hours, the median of the length of stay in the hospital after surgery in the obese group was 2 (2, 2) days, the number of macrosomia in the obese group was 8 (12.7%) and the number of the neonates who were admitted to NICU in the obese group was 25 (39.7%). Although a higher percentage of PPH occurred in the obese group (5, 7.9%) compared to the normal weight group (3, 4.7%), there was no statistical difference between them.

**Table 2:** Postoperative outcomes among pregnant women based on body mass index category, mean ± SD, median (Q1, Q3), and n (%) ^&^chi-square test; ^#^Kruskal-Wallis test SD: standard deviation; Q1: first quartile; Q3: third quartile; BMI: body mass index; n: number

Characteristic	BMI				p-value^&^
	Normal weight (n = 64)		Overweight (n = 79)	Obese (n = 63)	
Postpartum hemorrhage n (%)	64 (100.0%)	79 (100.0%)		63 (100.0%)	0.753
No	61 (95.3%)	74 (93.7%)		58 (92.1%)	
Yes	3 (4.7%)	5 (6.3%)		5 (7.9%)	
Macrosomia n (%)	64 (100.0%)	79 (100.0%)		63 (100.0%)	0.030
No	63 (98.4%)	75 (94.9%)		55 (87.3%)	
Yes	1 (1.6%)	4 (5.1%)		8 (12.7%)	
Initial time of mobility from bed (hours), median (Q1, Q3)	6 (5, 6)		6 (6, 7)	7 (7, 9)	< 0.001^#^
Initial time of intestinal sounds (hours), median (Q1, Q3)	9 (8, 10)		10 (8, 12)	10 (8, 14)	0.026^#^
Duration of surgery (minutes), median (Q1, Q3)	35 (30, 40)		35 (30, 40)	45 (40, 60)	< 0.001^#^
Initial time of starting breastfeeding (hours), median (Q1, Q3)	2 (1, 2)		2 (1.5, 5)	8 (4, 12)	< 0.001^#^
Length of stay in the hospital after surgery (days), median (Q1, Q3)	1 (1, 1)		1 (1, 1)	2 (2, 2)	< 0.001^#^
Admission to NICU n (%)	64 (100.0%)	79 (100.0%)		63 (100.0%)	0.001
No	59 (92.2%)	67 (84.8%)		38 (60.3%)	
Yes	5 (7.8%)	12 (15.2%)		25 (39.7%)	

According to the results, the overall prevalence of GDM in the research groups was 30 (14.6%), and GDM increased as BMI increased. Compared to the normal group, the obese group had a considerably greater prevalence of GDM (15, 23.8%), with an odds ratio (OR) of 3.02 (Table 3). The results also demonstrated a significant correlation between rising BMI and the prevalence of gestational hypertension (88, 42.7%). When comparing the obese study group to the normal BMI groups, the OR for gestational hypertension was 2.38 (Table 3).

**Table 3:** Odds of GDM and gestational hypertension diseases among pregnant women based on body mass index category GDM: gestational diabetes mellitus; BMI: body mass index; OR: odds ratio; n: number Simple logistic regression is used

Variable	GDM (yes)	p-value	OR (95% CI)	Gestational hypertension (yes)	p-value	OR (95% CI)
BMI category, n (%)	30 (14.6%)	0.080	-	88 (42.7%)	0.023	-
Normal weight	6 (9.4%)	-	1	23 (35.9%)	-	1
Overweight	9 (11.4%)	0.696	1.24 (0.42, 3.70)	29 (36.7%)	0.924	1.03 (0.52, 2.05)
Obese	15 (23.8%)	0.034	3.02 (1.09, 8.39)	36 (57.1%)	0.017	2.38 (1.16, 4.85)

Finally, the results showed that, as a whole, 42 (20.4%) of the three study groups had neonatal NICU admissions, and the rate increased as BMI increased (p = 0.001). With an OR of 7.76, the prevalence of neonatal NICU admissions in the obese group (25, 39.7%) was significantly higher compared to the normal weight group (Table 4).

**Table 4:** Odds of admission of neonates to NICU based on the body mass index category of the mothers BMI: body mass index; NICU: neonatal intensive care unit; n: number; OR: odds ratio Simple logistic regression is used

Variable	Admission to NICU n (%)	p-value	OR (95% CI)
BMI category, n (%)	42 (20.4%)	0.001	-
Normal weight	5 (7.8%)	-	1
Overweight	12 (15.2%)	0.182	2.11 (0.70, 6.35)
Obese	25 (39.7%)	<0.001	7.76 (2.73, 22.03)

## Discussion

The accumulation of body fat is a feature of obesity, and women with a BMI ≥ 30 kg/m² are considered to be at risk for developing chronic diseases and having challenges with giving birth, like stillbirth and miscarriages [11], as well as neonatal complications [12]. The present study was designed to compare maternal and neonatal outcomes between obese and non-obese pregnant women who underwent C/S in Basra city in the south of Iraq. Of the pregnant women in our study, 142 (68.93%) were older than 30 years old and had a BMI higher than 25 kg/m².

Baseline characteristics of pregnant women based on BMI category

According to the current study, the results show that the majority of mothers who were overweight or obese had low levels of education compared to the normal weight group. This issue could be explained by the fact that highly educated women are more likely to maintain a healthy lifestyle, which includes regular exercise and a balanced diet, and to be concerned about their weight and body shape. Low educational attainment may also contribute to inadequate use of medical care during pregnancy, according to another hypothesis [13]. Our results agreed with the scientific study published by Shah et al. (2018) [5], and with a hospital-based cross-sectional study in Iraq by Al-Kubaisy et al. (2014) [14].

Housewives made up the largest occupation among the study groups, counting 191 of 206 (92.72%). It was 56 of 64 (87.5%) in the normal weight group, 74 of 79 (93.7%) in the overweight group, and 61 of 63 (96.8%) in the obese group. This finding is also consistent with a population-based study conducted in Kuwait [15] as well as an observational cross-sectional study conducted in Indonesia under the direction of Yunanto et al. [16] that found housewives were more likely to be obese.

It is well known that overweight, obesity, and severe obesity increase the risk of hypertension and tachycardia, especially during pregnancy, and this thing is still supported by our study. In this study, the incidence rate of C/S increased in obese women compared to non-obese women. Similar to our results, studies [17,18] have also shown that obese women are much more likely to give birth by C/S. The main reason why obese women have more C/Ss than non-obese women is due to decreased myometrial function, which results in fewer and weaker contractions [19].

Comparable to other studies, the current study's results demonstrated a correlation between women's higher parity and higher BMI and obesity. An Iranian cross-sectional study under the leadership of Taghdir concluded that obesity and increased parity were positively correlated in a statistically significant way [20,21]. Additionally, a major population-based study [21] revealed that women having at least a single pregnancy had a higher average BMI than those who had never given birth.

Our study also showed that stillbirth is significantly associated with an increased BMI. Previous studies have shown similar results [22,23].

The findings of this study also demonstrated a strong correlation between obese pregnant women and a number of problems, such as a higher incidence of gestational diabetes and gestational hypertension. This result is consistent with a recently published retrospective cross-sectional study by AlAnnaz et al. (2024), which found that maternal obesity has a substantial impact on mothers' and newborns' immediate and long-term health outcomes, increasing the risk of complications like gestational diabetes and C/S deliveries [24]. Furthermore, a study conducted in 2016 by Al-Hakmani et al. found that mothers who are overweight or obese had a higher risk of gestational diabetes and hypertension than mothers who are normal weight [25]. In addition, Savitri et al. (2016) also revealed that the risk of gestational hypertension increased by 6% for every 1 kg/m² increase in BMI [26].

Postoperative outcomes among pregnant women based on BMI category

Maternal Outcomes

Postpartum hemorrhage: Our results showed that the number of mothers with PPH in the obese and overweight group was higher compared to the normal weight group in spite of the fact that there wasn’t a statistical difference among them. These findings are similar to the results of a comparative cross-sectional study by Rashid et al. (2022), who found that PPH in obese compared to non-obese groups was the highest despite the non-significant statistical difference between the two groups [11].

Duration of surgery: The result of the present study showed that obese mothers have a longer duration of surgery than overweight and normal-weight mothers. Our results are comparable with those published by Girsen et al. (2014) and Conner et al. (2014), who found that compared to women of normal weight, those who were obese or morbidly obese had lengthier operating times [27,28]. Additionally, our results concurred with an observational cross-sectional study under the leadership of Dabian in 2024, who concluded that BMI > 30 kg/m² was linked to a longer length of stay in the operating room [29].

Initial time of starting mobility: The results of the present study show that there is a significant difference between the obese group and the other two groups, where the obese mothers need a longer time to start mobilization after C/S.

Comparable to our results, studies [28,30] found that increasing BMI is associated with post-cesarean complications like wound complications, the incidence of deep venous thrombosis (DVT), endometritis, longer hospital stays, and pyrexia. These findings suggest that higher BMI is linked to increased postoperative complications, which can contribute to delayed initial mobility in obese mothers following C/Ss.

Initial time of return of intestinal sounds: Our results show that obese mothers have a longer time than the normal weight group regarding the return of bowel motion. These findings can be explained by the fact that obese mothers frequently need longer surgical procedures, higher dosages of anesthesia, and more opioids to treat their postoperative pain. They also have less mobility after surgery because of increased pain, wound complications, or insufficient physical activity, which can further delay the recovery of the intestines.

Loubert and Fernando, in a review study on anesthetic considerations for obese parturients, reveal that obesity is a significant risk factor for diabetes, which may cause gastroparesis and delayed gastric transit [31]. This delayed gastric transit can contribute to a longer time for bowel sounds to return postoperatively in obese women compared to their non-obese counterparts. Additionally, a review on C/Ss in morbidly obese patients highlights that these individuals face increased surgical, anesthetic, and logistical challenges, which can contribute to prolonged postoperative recovery times [32].

Initial time of starting breastfeeding: The results of the present study revealed that obese mothers have a longer initiation time of breastfeeding compared to normal weight. Our findings are comparable to a literature review study by Wojcicki, who concluded that the initiation and duration of breastfeeding can be negatively affected by increased BMI [33]. Also, according to a US analysis of medical data, women who were obese had a lower likelihood than women of normal weight of positioning the baby to the breast during the first two hours [34].

Length of stay in the hospital after surgery: This study showed that obese women (BMI ≥ 30 kg/m²) had longer hospital stays after surgery than overweight and normal weight groups, and this could be explained because of the longer duration of surgery as well as more received postoperative sedation and the need for more health care. Our results are in agreement with several studies. Saadia (2020), in a retrospective observational study, studied data collected from 245 pregnant women and revealed that the length of hospital stay increased as BMI increased [30]. Similar to our study, Rubens et al. (2022) [17] discovered that obese women had considerably higher hospitalization expenditures and prolonged stays compared to non-obese mothers.

Neonatal Outcomes

Macrosomia: Based on neonatal outcomes, the current study demonstrated that a higher BMI was associated with a higher chance of fetal macrosomia. The results we obtained support previous, specifically designed studies by Dabian et al. [29], Bracken and Langhe [22], and Dasgupta et al. [35] that found a connection between newborns with macrosomia and increased BMI.

Admission to the NICU: Neonates of obese mothers, as shown in our results, had a higher rate of NICU admission (25, 39.7%) compared to neonates of overweight (12, 15.2%) and normal-weight mothers (5, 7.8%). The difference was statistically significant, with a p-value of 0.001. It showed that neonates of obese mothers and overweight mothers have, respectively, 7.76 and 2.11 times the chance of being admitted to the NICU compared to normal-weight ones.

According to a retrospective cohort study of 648 pregnant women in Southeast Nigeria, a considerably higher percentage of obese women than healthy (normal weight) moms had admitted their newborns to the NICU [36]. These findings are consistent with the current study. Our results are also echoed by a comparative cohort study conducted on 200 gravid women divided equally into an overweight group and a normal weight group [37], which found that the neonates of the overweight women have a greater NICU admission rate (47, 47%) than those with the normal weight mothers (10, 10%).

Limitations and strengths

There are a number of limitations to this study, such as patients' lack of cooperation in answering the questionnaire and pregnant women's inability to obtain information both before and throughout pregnancy due to the community customs and traditions. Nonetheless, the current study is further strengthened by the fact that it is the only one to the best of our knowledge that examines the precise timing of bowel motion return with the onset of mobility following C/Ss among BMI groups.

## Conclusions

The study concluded that obesity has adverse effects on pregnancy outcomes for mothers and their infants. A low educational level is associated with a high BMI, leading to high risks of postoperative complications. Gestational diabetes and gestational hypertension are more frequent in obese women. Obesity has many more adverse effects during the postoperative period, such as the long initial time of starting mobilization, the long initial time of returning intestinal sounds, and the long initial time of starting breastfeeding for mothers. Also, obesity has a longer duration of surgery compared to overweight and normal weight groups. The neonates of obese mothers have a high prevalence of macrosomia and increased admission to the intensive care unit. Obese women should be considered high risk and recommended to maintain weight to reduce any complications after surgery. The neonates of obese mothers have a high prevalence of macrosomia and increased admission to the intensive care unit. Obese women should be considered high risk and recommended to maintain weight to reduce any complications after surgery.
